# Artificial intelligence as a tool for diagnosis in digital pathology whole slide images: A systematic review

**DOI:** 10.1016/j.jpi.2022.100138

**Published:** 2022-09-08

**Authors:** João Pedro Mazuco Rodriguez, Rubens Rodriguez, Vitor Werneck Krauss Silva, Felipe Campos Kitamura, Gustavo Cesar Antônio Corradi, Ana Carolina Bertoletti de Marchi, Rafael Rieder

**Affiliations:** a University of Passo Fundo, Passo Fundo, Rio Grande do Sul, Brazil; b DasaInova, Diagnósticos da América S.A., São Paulo, Brazil; c Pathology Institute of Passo Fundo, Rio Grande do Sul, Brazil

**Keywords:** Artificial intelligence, Pathology, Diagnosis, Whole slide images

## Abstract

Digital pathology had a recent growth, stimulated by the implementation of digital whole slide images (WSIs) in clinical practice, and the pathology field faces shortage of pathologists in the last few years. This scenario created fronts of research applying artificial intelligence (AI) to help pathologists. One of them is the automated diagnosis, helping in the clinical decision support, increasing efficiency and quality of diagnosis. However, the complexity nature of the WSIs requires special treatments to create a reliable AI model for diagnosis. Therefore, we systematically reviewed the literature to analyze and discuss all the methods and results in AI in digital pathology performed in WSIs on H&E stain, investigating the capacity of AI as a diagnostic support tool for the pathologist in the routine real-world scenario. This review analyzes 26 studies, reporting in detail all the best methods to apply AI as a diagnostic tool, as well as the main limitations, and suggests new ideas to improve the AI field in digital pathology as a whole. We hope that this study could lead to a better use of AI as a diagnostic tool in pathology, helping future researchers in the development of new studies and projects.

## Introduction

Digital pathology has become more popular in the past few years mainly due to the implementation of whole slide images (WSIs) scanners on a large scale in clinical practice, which was approved by the FDA in 2017.^16^ The use of these scanners allowed not only the adoption of remote work through telepathology but also the creation of large digital databases of pathology slide images.

Nowadays, the pathology field, as a medical specialty, faces the challenge of pathologist shortage due to lack of visibility of the profession, both in medical schools and among physicians.^42^ In the US, for example, there are around 21 000 active pathologists, and the trend is pessimistic: there was a decrease of 18% of American and Canadian pathologists between 2007 and 2017.^35^ However, pathology is an essential area for patient care, providing diagnosis in most of the diseases, including all types of cancers. This scenario also contributed to the need for growth of digital pathology.

Thus, digital pathology today faces 3 major fronts (Analog^3^), which must be solved with the growth of digitization and greater computational capacity for artificial intelligence (AI) algorithms: (1) Laboratory operations, with increased efficiency, quality control, and image management; (2) clinical decision support, with algorithms detecting areas of interest or performing specific diagnosis; and (3) research and development, with the discovery of new biomarkers,^15^ correlating image characteristics with prognostics,^45^ or transcriptomics.^30^ This review will focus on the second one, which is the main task of the pathologist in the clinical practice.

The support of clinical decisions by automated systems could lead to a better quality of diagnosis. There are studies already reporting systems that found cancer missed by pathologists,^40^ and increased performance and efficiency in terms of time and costs of the whole diagnosis process (e.g., with the possibility of systems discarding benign slides, as proposed by Campanella et al.^8^).

AI in digital pathology has been already applied before the use of WSIs. Older studies have demonstrated that AI and computer vision techniques could discriminate diseases in pathology images.^5^ However, these image datasets were mainly composed by previously selected region of interests (ROIs). This method requires pathologists selecting the areas of interest previously, making it very laborious and technically not possible to be implemented in the clinical workflow at the laboratory.

The popularization of WSIs scanners created databases of real-world scenario images in a pathology routine pipeline. Thus, the use of AI in WSIs has quickly become the focus of new studies. Applying AI models in WSIs is not easy and trivial compared with more common problems, such as ImageNet,^12^ mainly because of the nature of these images, which has millions of pixels due to the huge resolution needed to capture cellular level structures.^27^

Moreover, the understanding of the current best methods of AI and preprocessing steps in pathology, which datasets are available and what are the most common diseases that are being analyzed by the studies could help a lot researchers to apply better techniques, improving accuracies, and to decide the best diseases to apply in new studies.

Therefore, the aim of this study is to analyze and discuss all the methods and results in AI in digital pathology performed in WSIs through a systematic review. In this study, we will focus only on systems that perform diagnosis in WSIs, investigating the capacity of artificial intelligence as a diagnostic support tool for the pathologist in the routine real-world scenario.

This study will analyze the main techniques used in classification problems, the most used image preprocessing steps, which tissues and diseases are having more focus, the most used datasets, how the studies perform a final whole slide-level diagnosis, and, finally, how well these systems perform in terms of precision.

This study is organized as follows: Section 'Material and Methods' highlights the method applied for the systematic literature review; section 'Results' shows the results obtained from the selected studies; section 'Discussion' discusses and analyses paths regarding the digital pathology field in AI; finally, section 'Conclusion' presents the conclusions and future work.

## Material and methods

This research presents a systematic literature review (SLR) following Preferred Reporting Items for Systematic Reviews and Meta-Analyses (PRISMA) guidelines.^31^ The search was conducted through August 14, 2020.

This research had 5 key questions as follows:

(1) What are the most common diseases and tissues evaluated?

(2) What are the most common public datasets used?

(3) What are the most used techniques of artificial intelligence in WSI?

(4) What are the most accurate techniques and models used?

(5) How were the final slide-level diagnosis performed?

## Search terms

### Databases and criteria

The search expression was constructed to apply the most ranged search of AI (including the most used terms such as machine learning and deep learning) that were applied to pathology in whole slide images. The string was as follows: ("machine learning" OR "deep learning" OR "artificial intelligence") AND (pathology OR histopathology OR histopathological) AND (wsi OR "whole slide").

The study used multidisciplinary databases, from Computer Science and Health Sciences: Association for Computing Machinery (ACM), Institute of Electrical and Electronics Engineers (IEEE), PubMed, ScienceDirect, and Springer.

The search expression was limited by 8 terms because of a recent restriction of the Science Direct database and it was unmodified for all databases. The research considered all primary studies published until August 2020. All the studies that were not reviews were considered. Short papers were included because they are widely used in studies that describe new artificial intelligence algorithms due to the large use of annotated images of public datasets and challenges.^24^^,^^25^^,^^32^^,^^48^

Eligibility criteria for result inclusion in the final analysis are described below:

- EC1: Artificial Intelligence techniques applied as a diagnostic tool in pathology.*

- EC2: Validation of the model applied in whole slide images.

- EC3: Hematoxylin and Eosin (H&E) stained slides.

- EC4: Paraffin sections stained.

- EC5: Final slide-level diagnosis was performed.

Therefore, the exclusion criteria were as follows:

- EXC1: Algorithms that validated the models in ROIs or patches, and not in the WSI.**

- EXC2: Studies that applied models in frozen sections or Tissue Microarrays (TMA).***

- EXC3: Studies applied to cytology (smear slides).

- EXC4: Immunohistochemistry (IHC).

- EXC5: Other stains (such as Giemsa, Gram, and Warthin-Starry).

- EXC6: Studies that were not a diagnosis itself, such as tumor segmentation or detection without subtype classification or tumor-infiltrating lymphocytes (TILs) detection.

*Artificial Intelligence was considered as a computer system that can perform a task that normally would require human intelligence. Thus, we considered any computational algorithm or technique that performed the task, regarding the other criteria.

**Studies that performed only a heatmap without using the patch-level classification to perform a final slide-level diagnosis were not included.

***Tissue microarrays were excluded even if they were in H&E stain and paraffin sections because it is not used for diagnosis, but for study or comparisons with IHC.^18^

Patch-level classification and tumor segmentations or detections without specific subtypes can be very useful in pathologist’s routine, assisting them to perform a more accurate and faster diagnosis. However, these approaches have not replicated a real-world scenario of a pathologist routine. The main objective of this review is to analyze the capability of AI to perform a final diagnosis, using a scanned whole slide image, as pathologists do. An AI capable of a high performance in these tasks can generate reliable final reports (in addition to creating heatmaps), which is a convenient way to improve the workflow.

### Selection process

The selection process was structured in 4 stages: Identification of studies, applying our search term in all databases; exclusion of duplicated studies and screening of studies by title and abstract as a preliminary application of the eligibility criteria; and evaluation with a full reading of the studies, applying all the criteria thoroughly.

Two researchers applied the selection process simultaneously and independently. Moreover, 3 other experts supervised the process, helping in the final evaluation of the studies and assisting in final decisions in divergent evaluations.

## Results

**Fig. 1:**
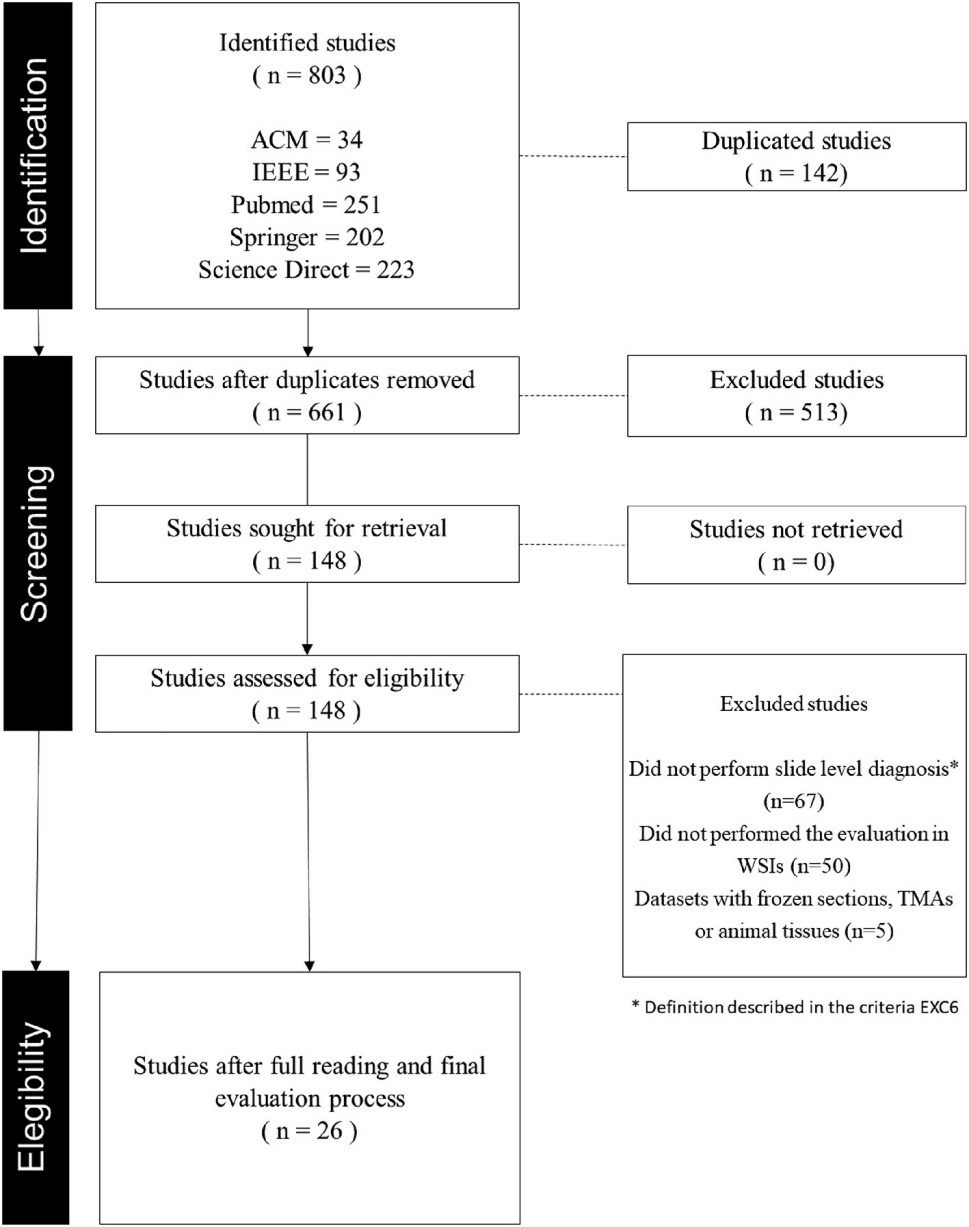
Selection process of the studies.

The SLR identified 803 studies in the surveyed databases using the search term adopted. At first, 142 duplicated studies were excluded. Another 513 studies were excluded by a screening of title and abstract, not presenting affinity with our eligibility criteria. Most of the oldest studies applied their validation on patches or ROI and another relevant part of the recent ones were studies in immunohistochemistry analysis, which both did not meet our eligibility criteria. Finally, a full reading and a careful evaluation of the remaining studies were performed. Another 122 studies were excluded, mainly because the vast majority either performed the final assessment in patches or performed detection/segmentation of cancer without the subtyping that is required in the pathologist's routine. Fig. 1 summarizes the pipeline.

Table 1 shows the final 26 studies that were selected. All of them performed a slide-level diagnosis, using the full WSI to perform the final inference. Also, all of them used H&E stain in paraffin-embedded sections. Detailed information of many columns of Table 1 can be accessed in Supplementary File 1.

**Table 1:** Summary of the studies in all aspects analyzed in this review.

Author	Year	Sample	Number of classes	*Diagnosis*^a^	*Dataset*^a^	Training set^b^	Test set	External test set	Pre-processing^a^	Model (Patch level)^a^	Model (Slide level)^a^	*Transfer learning*	Training approach	Results^a^	Results of the external test set^a^
Lucas et al.^33^	2019	Prostate	4	Cancer	Private	268 000 patches	89 000 patches	–	Data Augmentation	InceptionV3 + SVM	Percentages of GPs used for final Gleason grade	No	Supervised	Kappa: 0.70	-
Pantanowitz et al.^40^	2020	Prostate	18	Cancer	Private	549 WSIs	2501 WSIs	1627 WSIs	Tissue segmentation and data augmentation	InceptionV1, InceptionV3 and ResNet101	Maximum score	Yes	Supervised	AUC: 0.997^g^	AUC: 0.991, 0.941, 0.971, and 0.957^h^
Ström et al.^49^	2020	Prostate	2 and 4^a^	Cancer	Private	1069 WSIs	246 WSIs	73 WSIs	Tissue segmentation and data augmentation	30 InceptionV3 models	Boosted tree	Yes	Supervised	Kappa: 0.83	Kappa: 0.70
BenTaieb et al.^6^	2017	Ovary	5	Cancer	Public	68 WSIs	65 WSIs	–	–	K-means	LSVM	Yes	Weakly Supervised	Kappa: 0.89	–
Barker et al.^4^	2016	Central nervous system	2	Cancer	Public	302 WSIs	45 WSIs	302 WSIs	Tissue segmentation, color deconvolution and nuclei segmentation	–	Feature Extraction + Elastic Net (Regression)	No	Weakly supervised	Accuracy: 1.0	Accuracy: 0.93
Xu et al.^60^	2017	Central nervous system	2	Cancer	Public	55 WSIs	40 WSIs	-	Tissue segmentation, resize and data augmentation	Customized AlexNet	Feature Pooling + SVM	Yes	Supervised	Accuracy: 0.975	–
Bulten et al.^7^	2020	Prostate	7	Cancer	Private	933 WSIS	210 WSIs	–	Tissue segmentation and data augmentation	Own CNN to detect tumor and U-Net to final label	Normalized percentage of the volume of each class	No	Supervised (with a semi-automatic annotation)	Kappa: 0.819 on Gleason score	–
Gecer et al.^17^	2018	Breast	5	Cancer	Private	180 WSIs	60 WSIs	–	Color Normalization	RoI detector and an own proposed CNN	Majority voting	No	Weakly supervised	Accuracy: 0.55	–
Silva-Rodríguez et al.^46^	2020	Prostate	4 and 1^a^	Cancer	Public	155 WSIs	2122 patches	-	Tissue segmentation and data augmentation	Own CNN	MLP	No and yes^a^	Supervised	Kappa: 0.732	–
Tokunaga et al.^53^	2019	Gastric	4	Cancer	–	29 WSIs	–	–	Data augmentation	AWMF-CNN	Aggregating CNN	No	Supervised	IoU (Mean): 0.536	–
Sali et al.^43^	2019	Small intestine	4	Celiac disease	Private	336 WSIs	120 WSIs	-	Tissue segmentation, color normalization, resize and data augmentation	Customized Resnet50	Sum of all labels and majority	No	Weakly Supervised	Accuracy: 1.0	-
Xu et al.^61^	2020	Prostate	3	Cancer	Public	312 WSIs	49,883 patches	–	Grayscale and tissue segmentation	Feature extractor	PCA and SVM	No	Weakly Supervised	Accuracy: 0.771	–
Mercan et al.^34^	2018	Breast	14	Cancer	Private	240 WSIs	60 WSIs	–	–	Feature extractor + Linear classifier	PCA and SVM	No	Weakly supervised	Average precision: 0.737	–
Adnan et al.^1^	2020	Lung	2	Cancer	Public	1026 WSIs	–	–	RoI selection	Feature extractor	GCN	No and yes^a^	Weakly supervised	0.89 AUC^f^	–
van Zon et al.^56^	2020	Skin	3	Cancer	Private	232 WSIs	331 WSIs	-	Tissue segmentation and data augmentation	U-Net	Own CNN	No	Supervised	0.954 Accuracy^d^	–
Wang et al.^57^	2019	Lung	4	Cancer	Private	754 WSIs	185 WSIs	-	Tissue segmentation, resize and data augmentation	ScanNet	Aggregation of patch preditcions values + Random forest^a^	No	Weakly supervised	Accuracy: 0.973	–
Syrykh et al.^51^	2020	Lymph node	2	Cancer	Private	75% of 378 WSIs	25% of 378 WSIs	48 Cases	Tissue segmentation	CNN^a^	Average of patch inferences	–	Weakly supervised	AUC: 0.99	AUC: 0.69
Wei et al.^58^	2019	Small intestine	3	Celiac disease	Private	1,018 WSIs	212 WSIs	-	Data augmentation and color normalization	ResNet50	Threshold to discard low confidence + Most frequent predicted class	Yes	–	Average F1 score: 0.872	–
Korbar et al.^28^	2017	Small intestine	6	Colorectal polyps	Private	458 WSIs	239 WSIs	-	Data augmentation, color normalization and resize	ResNet-D	At least 5 positive class patches with 70% of confidence	No	Supervised	Overall F1 score: 0.888	–
Nagpal et al.^37^	2019	Prostate	4	Cancer	Public and private	1,226 WSIs	331 WSIs	-	Data augmentation	Customized inception V3	K-nearest neighbor model from patch prediction	No	Supervised	Gleason Score Accuracy: 0.70	–
Olsen et al.^39^	2018	Skin	3 models with 2 classes	Cancer	Private	Study 1: 300 WSIs	Study 1: 126 WSIs	–	Tissue segmentation	Derivative VGG + Rule-based discriminator	Classification model trained with the segmented areas^a^	No	Supervised	Study 1 Accuracy: 0.9945	–
						Study 2: 225 WSIs	Study 2: 114 WSIs							Study 2 Accuracy: 0.994	
						Study 3: 225 WSIs	Study 3: 123 WSIs							Study 3 Accuracy: 1.0	
Wei et al.^59^	2019	Lung	6	Cancer	Private	RoIs from 279 WSIs	143 WSIs	–	Tissue segmentation, data augmentation and color normalization	ResNet18	Threshold to discard low confidence + Most frequent predicted class	Yes	Supervised	Kappa Score: 0.525	-
Ianni et al.^20^	2020	Skin	4	Cancer	Private	85% of 5070 WSIs	15% of 5,070 WSIs	13,537 WSIs	–	Own Enconder-Decoder CNN + U-Net	Own CNN	No	Supervised (Patch) and Weakly Supervised (Slide)	–	Accuracy: 0.98
Iizuka et al.^21^	2020	Stomach & Small intestine	2 models with 3 classes	Cancer	Private	Stomach: 3,628 WSIs	Stomach & Colon: 500 WSIs	Stomach & Colon: 500 WSIs	Tissue segmentation and data augmentation	Customized Inception V3	RNN using the last but one layer from the previous model as input	No	Supervised	AUC e:	AUC e:
														Stomach: 0.97 and 0.99	Stomach: 0.98 and 0.93
						Colon: 3,536 WSIs								Colon: 0.96 and 0.99	Colon: 0.97 and 0.96
Campanella et al.^8^	2019	Skin	2	Cancer	Private	8387 WSIs^c^	1575 WSIs^c^	–	–	ResNet34	RNN using the last but one layer from the previous model as input	No	Weakly supervised	AUC: 0.994	–
Chuang et al.^9^	2020	Larynx, lip and oral cavity, esophagus, pharynx	3	Cancer	Private	626 Cases	100 Cases	–	–	ResNetXt	ResNet using the probability map as input	Yes	Supervised	AUC: 0.985	–

Captions – Not mentioned or not performed

a Details can be found in the Supplementary Table

b Training and validation set used during training was considered as training set in this column

c Not clearly specified, only the test set size and the whole dataset size, this number was estimated with these 2 information

d No metrics were performed by the authors in terms of final diagnosis, we calculated this metric using the table of misclassifcation comparison

e AUC of adenocarcinoma and adenoma compared to benign, respectively

f This study used the same model in 2 different tasks of lung carcinoma, one in a private set with 4 classes, and another in the TCGA differentiating 2 classes. We considered the most complex task.

g Authors performed only the Benign vs. Cancer AUC in the internal test set.

h Metrics representing: Benign vs Cancer, Gleason score 6 or ASAP vs Gleason score 7–10, ASAP or Gleason pattern 3 or 4 vs Gleason pattern 5, Cancer without vs with perineural invasion, respectively

### Samples and diseases

Notably, the majority of the studies performed AI in cancer (88.46%, n = 23). Only 3 studies focused on non-cancer problems, 2 of them in celiac disease,^43^^,^^58^ and 1 in colorectal polyps.^28^ Prostate cancer (n = 7) is the highest focus in pathology and AI, followed by skin cancer (n = 4).

### Datasets

Regarding data sources, most of the studies used their own datasets, even with public datasets being common.^24^^,^^25^^,^^32^^,^^48^ The most used public dataset was from TCGA,^38^ which has a large variety of cancers, with over 1.2 petabyte of data, including pathology slides. However, most of the public datasets are not in WSIs, or do not have a pixel level annotation by pathologists.^23^^,^^48^ Other datasets, such as Camelyon,^32^ have 1399 WSIs with tumor marking in metastasis, without subtyping, not configuring a diagnosis. Thus, it is expected that there would be greater use of own datasets.

In terms of dataset size for training, the largest one was undoubtedly from Campanella et al.,^8^ which used almost 10 000 WSIs in a weakly supervised approach of skin basal cell carcinoma detection. Ianni et al.^20^ performed the evaluation on the largest external test size, with 13 537 WSIs. Some studies that performed weakly supervised learning, which requires only the slide-level diagnosis and does not need manual annotations from pathologists also showed large datasets. For example, Adnan et al.^1^ and Wang et al.^57^ used 1026 WSIs and 939 WSIs, respectively.

In supervised learning approaches, large datasets were also found, such as those from Ianni et al.^20^ and Iizuka et al.,^21^ with 18 607 WSIs and 9164 WSIs, respectively. Other approaches, such as semi-automatic annotation,^7^ and training in ROI areas, and performing inference in the WSIs,^39^ were also used.

### Preprocessing

On preprocessing approaches, the only one that was used in all studies, was to divide the WSI into smaller patches. This approach was already well discussed by Komura and Ishikawa,^27^ and is a gold-standard method, mainly because of the low computational capacity of GPUs in terms of memory. Images that can have more than 10 billion pixels, cannot be used fully as an input of a neural network without overflowing memory. Therefore, all of the studies divided the WSIs into smaller patches (such as 256x256 pixels), using overlap or not as data augmentation, to feed their models.

Tissue segmentation was largely used as well (n = 14), mainly to avoid useless data. The most used technique was a simple threshold (n = 9). Other complex approaches, such as from Pantanowitz et al.,^40^ that used a Gradient Boosting to detect the background and blurry areas, or even as an output class of the classification model^7^^,^^9^ were also used.

Another technique that seems crucial in pathology is color normalization due to the high variability of the tissue staining process and scanners. Color normalization was used in 6 studies and was approached with different techniques, such as color deconvolution^63^ (n = 1) and normalization using the mean and standard deviation of the entire training set (n = 5).

Data augmentation was used in 15 studies, and the most common augmentations were rotations (n = 11) and flipping (n = 10), followed by color augmentations (n = 7) (e.g., color jitterings, random brightness, and contrasts, etc), Gaussian blurring (n = 2), resizes (n = 2), and translation (n = 1). Campanella et al.^8^ affirmed that in their large size dataset data augmentation did not seem to help in accuracy improvement in training evaluations.

Some studies used features extracted from the images (n = 4), mainly with nuclei segmentation techniques,^4^^,^^34^^,^^61^ but it seems to be obsolete machine learning approaches, that it may to be not ideal as deep learning to solve problems, which extracts the best features by itself.^29^ To corroborate this statement, some studies used machine learning models as feature extractors,^6^^,^^60^ tending to be a more practical and efficient method.

### Models and training approaches

In the pathology field, it is common to have 2 steps of classification, due to the patch-based approach to handling the gigantic size of the WSIs: one for the patch-level classification and another for the slide-level classification, using the patch-based classification as an input parameter.

In patch-level classification, deep learning models were largely used (n = 22), followed by a few older studies that used classical feature-based machine learning models (n = 4). In the classical machine learning approach, with manual feature extractions being done before model training, the most used feature extractors were described in the previous sections. Automated feature extractors, using CNNs, were also used in some studies. For final classification, SVMs,^34^^,^^60^^,^^61^ GCNs,^1^ and even regression models were used.^4^

In deep learning, the most used models were the ResNets versions (n=7) (mainly the Resnet50) and the Inceptions (n = 5) (mainly the Inceptionv3). Many own proposed convolutional neural networks (CNNs) were also used (n = 4), or even modified versions from state-of-the-art models (n = 3). Notably, most parts of the studies did not present detailed comparisons of different models' accuracy during training or test.

In slide-level classification, many different approaches appeared, from using the most frequent class^39^^,^^58^ to using a second deep learning model with the features of the previous model as an input^8^^,^^21^ to output a slide-level prediction. Unlike from the patch level, it is not clear what is the most accurate and most used method for classification. Using a model to final classification is the most common method to a final slide classification (n = 17), but arbitrary values and thresholds using the prediction values of each patch had good results as well. In this topic, Ianni et al.^20^ showed a much better result using arbitrary values of threshold in final slide prediction.

Moreover, one of the methods that are certainly a peculiarity of digital pathology and AI is that almost half of the studies use weakly supervised learning to train their models (n = 10). This approach works around the problem created by patch-based classification because it does not need fine annotation by experts, which gives a class for each patch extracted from the WSI. In this way, the most common method was authors using only the label from the report in a slide level as the output of their network, as the same way from classic classification in other fields, such as radiology. There were also studies that also used coarse labelling in supervised approaches, with selection of ROIs to represent the slide diagnosis^28^^,^^39^ and even using the WSI viewer logs of pathologists during the diagnosis.^34^

### Evaluation metrics and limitations

In terms of results, great metrics were shown by many studies with different complexities. In binary tasks, Campanella et al.,^8^ Chuang et al.,^9^ and Syrykh et al.^51^ reported AUC values of 0.994, 0.985, and 0.99, respectively, and Olsen et al.,^39^ and Barker et al.^4^ reported all accuracy values over 99%. In multi-class tasks, Sali et al.^43^ reported 100% of accuracy, and Kappa values of several studies showed greater or even metrics of AI compared to pathologists.^7^^,^^37^^,^^39^^,^^49^

Pantanowitz et al.^40^ also reported the first missed cancer by pathologists that was detected by their algorithm. Some studies^4^^,^^6^^,^^60^ showed small test sets, and only a few studies^4^^,^^20^^,^^21^^,^^49^^,^^51^ used external test sets, which shows major limitations in pathology studies, meaning that most studies in this review are no longer following the CLAIM guideline,^36^ which reports the best practices needed for AI studies in health.

There was a high variability of metrics used in the studies. The most common were AUC, accuracy, F1-score, and kappa correlation. There have not been many studies that have reported ground-truth annotated by multiple pathologists over the same dataset, which is a known problem in healthcare and pathology as well.^55^

## Discussion

In this section, we will discuss all the same subsections reported in the results. We aim to create a complete discussion of all topics described previously and also bring attention to some limitations and possible paths to the future that could help the digital pathology field in AI.

### Samples and diseases

Regarding the choice of tissues and diseases for studies, there is a notable preference for cancers, with a much lower coexistence for other diseases. The choice is very understandable: cancer today affects a large part of the population, with the increasing need for accuracy and speed in diagnosis. In the healthcare field, being able to analyze biopsy characteristics with genetic predisposition or prognosis can be very useful in saving patients' lives and boosting cancer science.

In addition, in the field of computer science and machine learning, the use of extremely difficult cases and high intellectual capacity also drive and motivate machine learning researchers. The test of the capacity of finding patterns and learning of machines and models are evaluated.

However, it is important to emphasize that the vast majority of the volume of pathology laboratories is not oncology. In the majority, laboratories report many more cases of diseases such as gastritis, appendicitis, and cholecystitis. Thus, the lack of studies in easier and more common cases is a curious fact within digital pathology, after all, diagnoses that are easier to be distinguished in the image by humans must be perceived in the same way by the machine.

Assuming that accuracy in less difficult cases should be greater, releasing most of the pathologists' reporting volume in an area that is increasingly lacking physicians^35^ can be a fantastic solution for artificial intelligence in digital pathology, allowing experts to focus time and effort on difficult cases.

### Datasets

The use of proprietary datasets, at the expense of public datasets, is possibly caused by a lack of medical annotation and the only recent popularization of WSI scanners. Many public datasets are not in WSIs,^22^^,^^23^^,^^48^ and most of the TCGA data^38^ has no medical annotation. Partnerships with pathology labs can help with image annotation and offer fully scanned slides.

However, with the popularization of award-winning competitions platforms in the field of data science (e.g., Kaggle,^26^ DrivenData^14^) recent datasets have been with WSIs and medical notes.^24^^,^^25^^,^^32^ These methods may become popular, as everyone benefits: researchers and data scientists get free data to work with, and laboratories and entities receive the best possible solution among thousands of participants.

Moreover, competitions seem to have an important role in the medical field. In radiology, this topic was already discussed by Prevedello et al.^41^ which highlighted as the main contributions of the competitions the attraction of data scientists to the medical field, the sharing of new techniques and ideas, and, finally, a possible correlation between problems solved by competitions and the creation of commercial products in the real world. Pathology has fewer competitions, mainly due to the recent use of AI in pathology in general, but the creation of new competitions could lead to new problems being solved.

### Preprocessing

It is well known, and also a consensus, that one of the biggest challenges of digital pathology is the use of gigantic images (Komura, D., Ishikawa, S., 2018). In this case, this review points to what seems to be a global and unique solution: the division of images into small patches, with or without overlapping. It is important to note, however, that this limitation is purely due to the high computational demand of WSIs and machine learning models.

The patch division, in fact, tends to lag the analysis by the models, since the image is being “looked at” by the model only by a fragment of the whole. To get around this problem, some studies proposed the analysis by LSTMs,^62^ passing several fragments together, models with inputs with several different zooms,^54^ or initial search for ROIs (BenTaieb et al., 2019). Another common approach was to aggregate the results of each patch in a final slide-level diagnosis, using diverse approaches.

Data augmentation methods were very common. Unlike models for traffic resolution (detection of cars, license plates, pedestrians, etc.) for example, which will hardly have inverted data (car or pedestrian upside down), pathology cases can easily be rotated or inverted without loss or major change of information, due to the fact that cut and positioning of the biopsy on the slide is always random. Thus, the use of data augmentation techniques, especially with flipping, rotation, and overlapping, is easy to understand.

However, Campanella et al.^8^ reported in their comparison that the use of data augmentation techniques showed no relevant increase in performance. It is important to emphasize that Campanella et al.^8^ used the largest dataset, from many different countries, with high variability and huge size (dozens of times larger than ImageNet dataset^12^).

Thus, it seems that data augmentation has an important role in AI models in pathology when the scientists do not have a great variability and a huge amount of data and can help to address dataset problems such as variability in stain and scanners, sections out of focus, excess of paraffin, pen markings, etc.

Another technique that was well described with great results by Chen et al.^10^ is the use of synthetic data as data augmentation in healthcare, including pathology. They used synthetic images generated by GANs within real images to train their model with a good improvement of accuracy avoiding the need of more manual annotation. This approach was not used in any article in this review and could be a good strategy in future studies. However, it is important to be careful with the use of synthetic generation of medical data, as something that does not correspond to reality can be generated. Real-world data, it might to be the best approach if a good size of data is available.

Regarding stains, few studies performed the change from color images to grayscale.^4^^,^^49^^,^^60^ In this sense, the choice is not so logical, after all, nuclei and cytoplasms are still distinct in black and white images. Talo et al.^52^ performed comparisons between architectures and input colorations and did not notice much difference in identical architectures with color and black and white images, despite a slight advantage for color. Notably, RGB images can also use transfer learning from many models that were pre-trained on ImageNET,^12^ which uses colored images.

However, it appears that using grayscale shifting can be useful: it does not overly impact accuracy, but it does decrease the size of a model's parameters, making it lighter. This can lead to greater use of inference with good accuracy, especially in pathology huge images, which requires a lot more computational cost during inference.

In relation to tissue segmentation, complex techniques do not seem to be very useful: the use of thresholds of segmentation (thresholding) shows high performance, as the background is usually white, and the tissues are predominantly pink (hematoxylin) and purple (eosin). Nevertheless, Pantanowitz et al.^40^ showed good use of a complex technique in detecting background: using it as an out-of-focus detector as well.

Another problem faced by histopathological slides is the high color divergence, whether due to the application of stains, due to the physical–chemical process being carried out by a human, or due to the use of different scanners with different optical lenses. This problem was overcome with color normalization, using common techniques, such as normalization by histograms, or more elaborate techniques, such as color deconvolution.^63^ Color normalization is an important step in the process, and has been widely used in studies.

### Models and training approaches

The comparison of architecture and results in this review is actually limited by one topic that will be discussed in section 2.5.6: most of the studies in this review that used state-of-the-art models have not presented comparisons. Thus, comparative studies bring an advantage to new studies, as they compare architectures with the same learning rate parameters, the same database, and the same medical note, indicating the best architectures to use in similar diseases and tissues.

Another limitation on comparing architectures in a better way is due to the huge variability of datasets, in tissues, the complexity of diagnosis, and, mainly, in size. Studies that trained their models in larger datasets showed the best results of this review, which is expected in deep learning that is becoming more and more data-centric.

Firstly, the classical machine learning approach was observed in older studies (^1^^,^^4^^,^^34^; Xu, et al., 2020). The use of manual feature extraction before the model training had good results in these older studies, at least for the problems that were aimed to be solved by them. This indicates that the use of classical features that pathologists pay attention to, such as nuclei formats, size, and shapes, was a good discriminator for those diseases.

However, the use of manual feature extractions can be useful for a model, but it is not guaranteed. Deep learning models are better in selecting the most discriminant features for each problem and in pathology are not different, with some studies using deep learning to discover new biomarkers that can be used to discriminate diseases.^15^ Also, the use of deep learning models was always the top-performing in classical image classifications problems, and the same occurs in pathology when we analyze the top models in Kaggle’s pathology problems.^24^^,^^25^

In turn, deep learning models bias the use of state-of-the-art. These models have pre-trained parameters, making training much faster and lighter and they also have proof of effectiveness in giant general-purpose datasets like ImageNET.^12^ They also do not entail the need for modeling and engineering to create your own architectures.

Moreover, the use of state-of-the-art models seems to be effective with a large database, as the studies with the highest accuracy in this review used Inceptions and ResNets to a great extent.^8^^,^^9^^,^^21^^,^^40^^,^^49^ This is possibly due to the possibility of using pre-trained weights, greatly reducing training time and computational cost.

However, studies that performed comparisons between state-of-the-art and proprietary models indicate that this is not necessarily the best approach.^11^^,^^44^^,^^46^ Even more curious: simple models with a notoriously smaller number of layers and parameters showed good accuracy and precision. This was also noted even in comparative studies between state-of-the-art models, with models with smaller parameters showing better precision.^13^^,^^50^

Moreover, it seems that this is not an exclusive result for smaller datasets. Campanella et al.^8^ also showed better results with architecture with fewer parameters (ResNet34) compared to architectures like DenseNet101, VGG11BN, AlexNet, and ResNet101. Nevertheless, it is important to emphasize that newer architectures contain more complex structures, such as Inceptions and EfficientNets. The EfficientNet architecture, for example, had a surprising result in ImageNET^12^ because of their high accuracy with a lower number of parameters and might be a good architecture to be tested in future studies.

Another important factor that has gained the attention of researchers for increasing the performance of machine learning models is the use of model ensembles.^2^ The model ensemble is the multiple uses of inference models on the same data, using the same architecture, or different architectures, with different weights. This technique allows the machine to have different “looks” on the same data, and the average of its inference generates a much greater accuracy in the test data. This technique was rarely used in the studies of this review, appearing in both with high performance.^40^^,^^49^

Regarding the use of the training approach, supervised and weakly supervised methods were used equally. Supervised methods provide a more accurate look at the machine in areas of interest, however, they require expert notes, making it costly and leading to other problems, mainly the discrepancies between pathologists annotations and the diagnosis itself. In the studies by Bulten et al.^7^ and Ström et al.^49^ the Kappa correlation among the pathologists themselves was around 0.7.

One way to get around this, mainly in terms of cost, was the use of weakly supervised training, mostly with the technique of learning multiples by instances. In this technique, it is not necessary to annotate areas with the disease within the respective diagnosis, but only the final diagnosis of the slide. Thus, the fragments are all designated with the diagnosis class of the entire slide. The results were very promising with this technique, especially in the study by Campanella et al..^8^

The main difference between the 2 techniques is the volume of data. While the supervised one achieves optimal results with about 500 WSIs for training,^40^ in the supervised weaker optimal results were obtained with about 10 000 slides.^8^ Weakly supervised methods have the cost and time advantage of pathologists, with a higher computational cost for training due to a larger database, and supervised methods allow for a smaller database with greater accuracy, in theory. In practice, the divergence between pathologists in an annotation can be a problem for this methodology as well, although the same occurs between diagnoses of WSIs.

### Results and limitations

In the results, it is noticeable that the amount of data and the annotation quality is highly correlated to model precision. While the best results^8^^,^^20^^,^^21^^,^^40^^,^^49^ applied training in bases of more than 500 slides, less satisfactory results^17^^,^^59^ are obtained from not-so-large datasets.

Howard et al.^19^ alleges that the use of big datasets with high variability from multiple labs can lead to better results, allowing models to better discriminate the diseases, which cannot be true in site-specific studies, due to overfitting problems. Besides, the use of the same medical staff to annotate the training and test set can also overfit the models and bias the results.

In terms of the results with different training approaches, it is clear that the use of a supervised approach leads to better results with fewer data.^39^^,^^56^^,^^60^ However, the use of a weakly supervised approach also showed great results in huge datasets.^8^ The main problem with the weakly supervised approach was the need for huge datasets, which can be provided more easily, not requiring pathologist annotations, and binary problems to be solved, but Mercan et al.^34^ proposed a multi-class weakly supervised method with reasonable precision with an average size dataset.

Despite that, it is difficult to make comparisons between studies with such different methodologies. Therefore, it would be ideal for large studies to demonstrate the comparison of several architectures on top of the same database to provide support and evidence of why the methodology was chosen. It is noticeable, however, that this is not a simple task, given that the computational cost to analyze so much data can take days or even weeks.^8^ In any case, it would be ideal if a comparison was made on at least a portion of the data used.

This can be solved with a greater popularization of public challenges of deep learning in pathology, where the same dataset can receive several different methodologies and compare them in the results, besides being already demonstrably useful for a real scenario.^41^

In terms of limitations, few studies used external test sets, possibly biasing their results. This is a significant issue in the studies, since the models created can be overfitted in training data, stains, scanners, and population. The CLAIM guideline^36^ have a specific item for external test sets, considering as an essential practice for studies with reliable results.

Syrykh et al.^51^ for example, reported a huge drop of AUC in the external test set (0.99–0.69). This issue also could be solved with popularizations of public datasets from different labs and different countries. Of course, this requires great attention to ethical problems as well discussed by van der Laak et al.^55^ Errors in calculation of AUC metric were also found, where the authors used the binary prediction to generate the ROC curve.^1^

Moreover, despite an academic consensus on certain approaches to color treatment or data augmentation,^47^ many studies did not perform color normalization or data augmentation, for example, and most did not use model ensembles for inference, probably due to the high computational cost during inference when using multiple models.

To solve this problem, the method just proposed by Allen-Zhu and Li^2^ with self-distillation, which appears to bring the same ensemble results in the inference, may also be an outlet for new studies, especially for digital pathology, where the cost of both training and inference is a problem to be faced.

## Conclusion

This study aimed to present results of a systematic review of the literature on studies that applied artificial intelligence techniques for histopathology diagnoses in whole slides images. In this study, we sought to analyze, compare, and discuss the main points that involve the methodological process (database, image preprocessing, AI models, and training techniques) and also the results and limitations.

Furthermore, we proposed great attention to some studies limitations, such the poor use of external test sets and the lack of model comparisons. We also draw attention for the need for growing public datasets and competitions, as well as the use of self-distillation techniques to lead better results and fast inferences.

We hope that this study could lead to a better use of AI models for pathology as a diagnostic tool and help future researchers in the development of new studies. In addition, this review opens up new avenues of research for other diseases and allows summarized access to different techniques in different approaches, in different tissues, and different diseases.

## Declaration of interests

The authors declare that they have no known competing financial interests or personal relationships that could have appeared to influence the work reported in this paper.

## CRediT authorship contribution statement

**João Pedro Mazuco Rodriguez:** Conceptualization, Methodology, Writing – original draft, Investigation. **Rubens Rodriguez:** Conceptualization, Methodology, Investigation. **Vitor Werneck Krauss Silva:** Investigation, Data curation, Writing – review & editing. **Felipe Campos Kitamura:** Investigation, Writing – review & editing. **Gustavo Cesar Antônio Corradi:** Investigation. **Ana Carolina Bertoletti de Marchi:** Writing – review & editing. **Rafael Rieder:** Methodology, Writing – review & editing, Supervision.

## Appendix A Supplementary data

Supplementary material Image 1
